# Ending an Odyssey? The Psychosocial Experiences of Parents after the Genetic Diagnosis of a Mitochondrial Disease in Children

**DOI:** 10.3390/jpm14050523

**Published:** 2024-05-14

**Authors:** Oliver Heath, Emma Hammerl, Anna Spitzinger, Saskia B. Wortmann

**Affiliations:** 1 University Children’s Hospital, Salzburger Landeskliniken (SALK), Paracelsus Medical University (PMU), Müllner Hauptstrasse 48, 5020 Salzburg, Austria; o.heath@salk.at (O.H.); e.hammerl@salk.at (E.H.); a.spitzinger@salk.at (A.S.); 2 Institut für Klinische Psychologie der UK für Psychiatrie, Psychotherapie und Psychosomatik der PMU, 5020 Salzburg, Austria; 3 Nijmegen Centre for Mitochondrial Disorders (NCMD), Amalia Children’s Hospital, Radboudumc, 6525 Nijmegen, The Netherlands

**Keywords:** mitochondrial diseases, genetic diagnosis, value of a diagnosis, diagnostic odyssey, parental attitudes

## Abstract

Obtaining a genetic diagnosis of a primary mitochondrial disease (PMD) is often framed as a diagnostic odyssey. Yet, even after receiving a diagnosis, parents of affected children experience ongoing therapeutic and prognostic uncertainty and considerable psychosocial challenges. Semi-structured interviews (N = 24) were conducted with parents of 13 children (aged 2–19 years) with a genetically confirmed PMD. Paternal (N = 11) and maternal (N = 13) perspectives were obtained, and thematic analysis was performed on all interviews. A genetic diagnosis was valuable and empowering for parents, despite eliciting varied emotional responses. While the diagnosis helped focus management decisions, families often felt overwhelmed and unsupported in navigating the healthcare system. Most parents reported a serious impact on their romantic relationship. The sources of social support varied, with a preference for established friendship and family support networks over disease-specific community support groups. Most parents favored prenatal genetic testing in the event of a future pregnancy. This study provides insight into the lived experiences of parents after a genetic diagnosis of PMD in their children. The findings draw awareness to supportive care needs and highlight important gaps that should be addressed to ensure that parents feel supported within a holistic framework of management for PMDs.

## 1. Introduction

Primary mitochondrial diseases (PMDs) are individually rare but as a group represent the most common form of inherited neurological diseases, with a prevalence of 1 in 5000. Currently, more than 375 different mitochondrial diseases are known, all sharing an underlying dysfunction in the mitochondrial oxidative phosphorylation system. PMDs vary substantially in their genetic background, age of onset, severity, and individual course of disease, but they are generally progressive and often lead to death before adulthood (for reviews see [1,2]).

The broad genotypic and phenotypic spectrum of PMDs poses challenges to their diagnosis, a journey that is often long and burdensome for families. One US–American study reports that, on average, a patient is seen by more than eight clinicians before the diagnosis of a PMD is made. Despite the enormous improvement in diagnostic yield attained with genomic testing, more than 50% of all patients suspected of having a PMD do not reach a genetic diagnosis [3,4,5]. Families’ experiences are often further compounded by the attribution of numerous other diagnoses that later turn out to be incorrect, which makes for an emotional rollercoaster on the way to the final (genetic) diagnosis [6]. Indeed, the metaphor of diagnostic odyssey aptly describes the often long and emotionally burdensome journey that these parents face in seeking a diagnostic explanation for a challenging medical problem in their child [7].

Next-generation sequencing techniques have revolutionized diagnostic possibilities and are paving the way to timelier diagnoses of genetic conditions [5]. Parents of children with rare genetic diseases who underwent exome sequencing indicated that a conclusive diagnosis had a positive impact on disease acceptance and coping with feelings of guilt [8]. Despite the absence of a specific treatment, parents reported decreased worries after receiving a definite/likely diagnosis for their child’s condition through exome sequencing [9]. In children with an intellectual disability, the value that parents place on receiving a genetic diagnosis has been categorized into the following seven areas: validation (that something is wrong with their child), information about the disease and its expected course, procuring services (e.g., in school), early intervention/management, support by family/friends/patient support groups, “need to know” (curiosity) and prenatal diagnosis [10]. These studies underscore the emotional utility that a diagnosis can provide beyond its clinical utility [11].

Understanding caregiver burden is important for natural history studies to describe the overall effects of a rare disease [12]. Parents caring for children with ultra-rare diseases often feel burdened by their children’s health problems and emotional and behavioral changes, and they may experience considerable stress and dissatisfaction with healthcare services [13]. Compared to the number of clinical and biochemical publications pertaining to mitochondrial disease, few studies have explored the needs and challenges of families living with mitochondrial disease, and all of them predominantly focus on maternal perspectives [12,14,15,16,17,18,19]. Mothers of children with mitochondrial disease reported significantly greater socioeconomic and psychoaffective strain and also required more healthcare resources compared to mothers of children with Phenylketonuria (PKU), another inborn metabolic disease; however, this is a disease for which successful treatment is available and lined out in guidelines [14]. Similarly, a Korean study found that mothers of children with mitochondrial disease were more likely to have greater caregiver burden and poorer quality of life than mothers of children with intractable epilepsy [17]. In this article, we present findings from interviews with parents (mothers and fathers) of children with PMDs, with the view to exploring the perceived value and impact of securing a genetic diagnosis of a PMD.

## 2. Materials and Methods

Participants in this study were recruited from the METAB-ERN recognized center of expertise for mitochondrial diseases at the University Children’s Hospital Salzburg (SALK), Austria. This study received ethical approval from the ethics committee of the Land Salzburg (EK Nr: 1036/2022). We adopted a qualitative approach underpinned by a phenomenological framework, with the aim of understanding parents’ lived experiences [20].

### 2.1. Participant Recruitment

**Figure 1:**
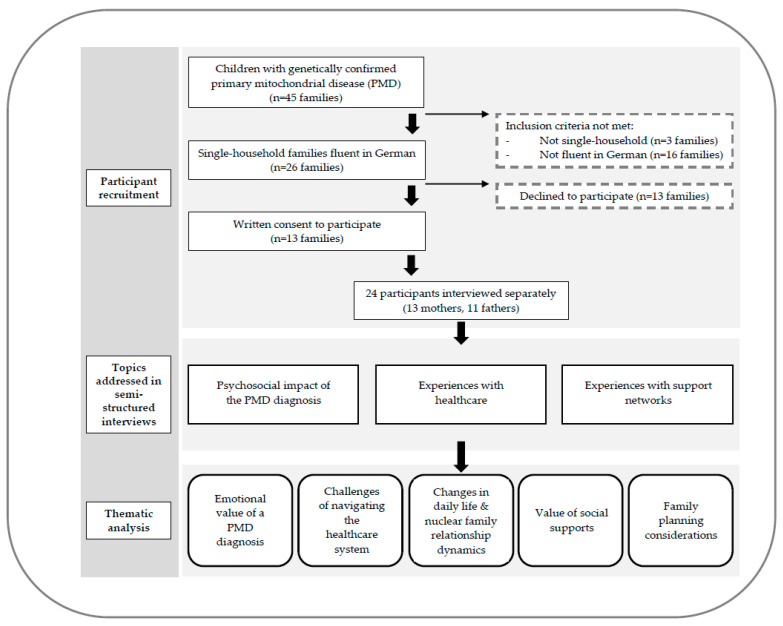
Conceptual overview of study design and results.

Parents of children diagnosed with PMDs were recruited using purposive sampling. Potential participants were identified through SALK hospital patient records. Suitability to participate in this study was assessed based on the following eligibility criteria: (i) parents of children with a genetically confirmed PMD (ii) living in a single household since the child’s birth and (iii) both fluent in German. A total of 26 families were found to be eligible. During the recruitment phase, families were first informed about the study during their child’s routine outpatient clinic appointment. An invitation letter further detailing the aims of the study was sent in an email to all eligible participants. Participants were given the option to request more information, consent to participate, or decline further contact. Families were not offered any incentives, financial or otherwise, to participate in the study. Ten families declined to participate, and thirteen parent-couples provided written informed consent to be interviewed (Figure 1). Two of the fathers subsequently pulled out prior to the scheduled interview due to competing family commitments.

### 2.2. Data Collection

Semi-structured interviews were held face-to-face with each parent separately, between July 2022 and March 2023. To mitigate potential response bias, participants were free to determine the location of the interview, be it in their home, the hospital clinic, or a neutral alternative. Most interviews were carried out in the participants’ homes. Interviews were conducted in German by a trained researcher (EH) who started by reiterating the aims of the study and providing reassurance that there were no right or wrong answers to the questions being asked. The interviewer guided the topics but left room for more elaboration and asked follow-up questions if clarification was needed. Interviews ranged in length from 60 to 90 min and were audio-recorded, transcribed verbatim, and de-identified. Open-ended questions focused on demographic characteristics and parents’ psychosocial experiences following their child’s diagnosis (Figure 1 and Supplementary Material Table S1).

### 2.3. Data Analysis

Transcripts of parental responses were analyzed and summarized using thematic analysis [21]. Researchers EH and OH independently drafted themes from the transcripts of the interviews. EH proposed the first category system, which was revised and rewritten by OH. The final thematic categorization was discussed with SBW to reach an agreement. Illustrative quotes were also extracted from the transcripts and translated from German. Transcript data were managed using Microsoft Office 2016 (16.0.5435.1000), and recordings were erased once no longer required.

## 3. Results

### 3.1. Demographic Characteristics

We completed interviews with families of 13 children, each affected by a genetically confirmed PMD (Table 1). The cohort of parents comprised 13 biological mothers and 11 biological fathers, whose median ages were 44 years (range: 33–54 years) and 46 years (range: 35–57 years), respectively. Ten respondents (42%; five mothers and five fathers) had apprenticeship formations, and eight (38%; five mothers and four fathers) had university degrees. Seven families had annual household incomes of EUR 60,000 or more. All families had one child with mitochondrial disease living at home, and the children’s median age at the time of enrolment was 9 years (range: 2–19 years). Most children (10/13) had nuclear gene defects. Moderate-to-severe global developmental delay or intellectual disability was reported in 9/13 children by 13/24 respondents. The median age of symptom onset was 20 months (range: 0–15 years), while the median age of genetic diagnosis was 4 years (range: 0–19 years). The median diagnostic odyssey duration reported was 18 months (range: 1 month–19 years). The timing of the interviews relative to the date of diagnostic disclosure varied widely, from 0 to 7 years after diagnosis (median 2 years).

### 3.2. Common Themes of Parents’ Lived Experience following a Genetic Diagnosis

We identified five common themes to classify parents’ experiences: (1) the emotional value of a PMD diagnosis, (2) challenges of navigating the healthcare system, (3) changes to daily life and nuclear family relationship dynamics, (4) the value of social support, and (5) impact on family planning (Figure 1 and Table 2).

#### 3.2.1. The Emotional Value of a PMD Diagnosis

Prior to their child’s diagnosis, all parents described feeling hopeful that symptoms would resolve over time. Diagnostic confirmation elicited a wide range of emotional responses, however.

Most parents (17/24) considered a diagnosis valuable, as this label provided validation that their child had a tangible condition. For some, this validation also extended to vindication, helping them overcome initial feelings of guilt.

*Mother: “[I felt] relief because it confirmed that I hadn’t actually done anything wrong”.*

*Father: “Before the diagnosis there was always the question: have we done something wrong because he’s so delayed? [...] Receiving a diagnosis allowed us to stop blaming ourselves”.*

The diagnosis also empowered parents (19/24) to process emotions they had been contending with. For some, this helped them move toward acceptance, while others also reported feeling less anxious and more confident to manage their child’s needs or to talk about the condition with friends and family (20/24).

*Father: “When the diagnosis wasn’t certain yet, I always just hoped that it was something that could be cured. At least with the diagnosis came the certainty that this was something serious that wouldn’t go away”.*

*Mother: “It was finally possible to label it, and with that, somehow, came a greater sense of security regarding everyday life and dealings with my child. Before that, everything was anxiety-ridden because I didn’t know what it was, how it would progress, how old she would live to, and I feared the worst. After learning about the diagnosis, everything became easier. There is still anxiety, but overall, there is less fear, and I am more hopeful again”.*

Half of the cohort (12/24) reported feeling immediate relief, as having a diagnosis helped to clarify expectations for the future.

*Mother: “Before knowing what this was, we would always worry about what if she has another episode, will she survive the next time, or how long will she live at all? The diagnosis provided some relief, and I felt more settled learning that affected children can live with this condition and grow older. Then there was no longer this fear that she might die at any time”.*

Among the rest of the cohort, genetic confirmation of a PMD for which there were no available treatment options was a blow to hopes for a “normal” child. Many also continued to worry about the variable prognosis and uncertainty that remained despite receiving a diagnosis.

*Mother: “The hope of life returning to normal was destroyed”.*

*Father: “Did knowing the diagnosis make me feel better in any way? I think that I would have felt some relief if I had been told that there was treatment available. But ultimately, I worried more after learning that my child has an unbelievably rare disease, the course of which can potentially be severe or life-threatening, and that children with this condition can die”.*

Overall, 73% of fathers (8/11) reported not feeling relieved by their child’s diagnosis, in contrast to 69% of mothers (9/13) who did.

#### 3.2.2. Challenges of Navigating the Healthcare System

Parents (19/24) felt empowered by their child’s diagnosis to seek out more information where possible and to make informed management decisions for the child and for their own well-being. Accordingly, participants reported that their child’s diagnosis justified eligibility for assistive devices and/or increased need for therapies, including early intervention and school support. Some parents (4/11) eventually sought psychological support for themselves, although most felt that the presence of a psychologist at the time of disclosure would have been of little benefit.

*Father: “Accessing the relevant therapies was not only important for the little one but also for my wife. It provided her with reassurance that she was doing the right thing, and with these supports in place she became more self-confident, and she worried less”.*

*Mother: “For a while, I accessed psychological support for myself too, to help address my own feelings of failure and guilt that she had got this disease from me. Fortunately, I realized relatively quickly that it was good to get help. It was important that I did that”.*

Nonetheless, parents also spoke to the challenges of navigating the healthcare system to advocate for their child’s needs effectively and access financial support. Overall, the majority (16/24) felt that even with a confirmed diagnosis, accessing supportive therapies for their child remained difficult financially and that a diagnosis did not fundamentally change their access to therapies or medical equipment. While some acknowledged the value of disability-related financial support, these subsidies were often perceived as insufficient, and most couples reported incurring substantial out-of-pocket expenses. These parents reported feeling “overwhelmed” at having to navigate the healthcare system themselves, with little support from social services. Their frustration was compounded by the scarcity of clear management guidelines and a perceived tendency of health insurance companies to underestimate the level of care and assistance required for the affected child.

*Mother: “The same applies to all parents of children with disabilities. How can I access more supports to help care for my child? Where is the balance between doing what is necessary, without this endeavor becoming all-consuming?”*

#### 3.2.3. Changes in Daily Life and Relationship Dynamics within the Nuclear Family

Most parents (16/24) reported that their child’s condition and caregiving needs did not preclude them from holding paid employment, nor did the distribution of caregiving activities change following the diagnosis. Among families interviewed, mothers often assumed most of the caregiver responsibilities and 10/13 (77%) also held concurrent part-time jobs, working on average 20.5 h per week (SD ± 6.4 h).

Perceived changes to the structure of daily life or financial aspects following a child’s diagnosis were split across the cohort, and to a small degree, these varied according to gender. Fifty-four percent (7/13) of mothers reported significant changes to their daily structure, while 63% (7/11) of fathers did not. Similarly, 54% (7/13) of mothers described a financial impact post-diagnosis, while 55% (6/11) of fathers responded otherwise. The changes described related to parents’ need to become more organized, having less time for respite activities and holidays, the toll of reduced employment hours, or having to take additional time off work.

More respondents (15/24) also acknowledged altered relationship dynamics within the nuclear family following their child’s PMD diagnosis. This often involved a shift in attention towards the needs of the affected child, sometimes at the expense of other siblings’ and parents’ own well-being (10/24).

*Father: “You don’t feel like doing anything else, other than looking after the little one and informing yourself about their condition. All attention is directed at the little one, not your wife, nor your other child, nor you for that matter. It’s all about the little one. [...] I’m just happy now that my little one is alive, but I always worry in the back of my mind about what will happen in two years, what will happen in three years?”*

*Mother: “My focus has shifted completely to him [affected child], and I feel as though I can only be happy when he gets well. How can I be happy when my child is so sick? I think it’s a pretty good description of changes in my functioning over the years since his diagnosis. That lightness of life is gone”.*

Higher levels of marital strain were highlighted by 13/24 respondents, as parenting roles took precedence, and participants acknowledged having less time and energy to invest in the parental couple relationship. Differences in the approach to their child’s illness or emotional processing styles, also contributed to perceived friction between parents in nine of the families interviewed.

*Father: “Things you used to do with your wife you no longer do. There is no more physical contact. Everything is focused on [our child’s] illness. Two parents fighting together for the child. Two fighters together, but no longer a couple; there is nothing that makes you say “yes, okay, we are still a couple””.*

Among those who had more than one child, 10/20 reported concerns about the adverse impact of a child’s diagnosis on the needs of unaffected siblings. Sibling reactions commonly reported included withdrawal, increased aggression, or negative comments towards family members.

*Father: “One day my oldest daughter, four years old, said to me: “I wish [my sister] wasn’t here”. It hurt a lot [to hear her say that], but it is totally understandable. She felt totally side-lined”.*

#### 3.2.4. The Value of Social Support

Most respondents reported receiving greater support and relief from family and friends (75% yes vs. 25% no) than from community support groups (50% yes vs. 50% no).

Interactions with extended family changed following the diagnosis, albeit to different extents for mothers and fathers. Sixty-two percent (8/13) of mothers described a change compared to 45% (5/11) of fathers. This involved either receiving greater support from the child’s grandparents (10/24), or conversely, reducing interactions with them due to perceived emotional strain, misunderstanding, or insensitivity on their part (10/24).

The majority (17/24) did not report significant changes in their interactions with friends post-diagnosis, although these interactions were generally limited to “true friends” who held space and supported them through their lived experience. More fathers viewed friendships as a valued source of distraction that helped them to cope with the burden of care. Those parents (7/24) who cited turning away from friends, did so due to perceived discomfort or because of their own time and energy constraints.

Contact with disease-related community support groups was not contingent on receiving a genetic diagnosis (20/24), and nine families reported being in contact with other families of children with disabilities. For most, interactions with other affected families occurred spontaneously at the hospitals, rehabilitation centers, or special needs institutions. Only five respondents had specifically reached out to other PMD-affected families after receiving a genetic diagnosis.

When asked whether they considered participation in PMD-related community support groups or contact with other affected families a source of relief, parents expressed a mix of opinions (12 yes vs. 12 no). Fifty-four percent (7/13) of mothers were in favor, while 55% (6/11) of fathers were against.

The rarity of individual PMDs and the wide spectrum of their presentations were often cited as barriers for parents to fully relate to other PMD-affected families, and 9/24 (five fathers, four mothers) reported finding it too confronting and sad to meet children who were more severely affected than their own child.

*Father: “I try to inform myself [about the condition] online, but I stopped seeking out support groups. My child has a milder form of the condition, and I felt out of place [in these groups] compared to others who are clearly more severely affected”.*

*Mother: “Of course, I would like to meet families of other similarly affected-children, children who have the same condition, and are of a similar age [to my child]. But that is rather unlikely and perhaps even unrealistic. That’s what is frustrating and difficult about such a rare disease, but you just have to live with it”.*

Others (9/24), however, valued interacting with families of children affected by chronic conditions other than PMD, citing a mutual understanding and shared experience of caring for someone with a disability. Discussion topics of importance related to available therapeutic services, concerns about the future, and a child’s evolving needs over time.

*Mother: “Seeing other [similarly affected] children, regardless of their diagnosis, and meeting other parents [in similar situations to ours], felt like a little cosmos. That was good and something we had not previously experienced at the regular kindergarten and school”.*

#### 3.2.5. Impact on Family Planning

Nearly all the parents interviewed (22/24) sought genetic counseling to understand the implications of their child’s PMD diagnosis on the risk of recurrence. The impact of a child’s PMD diagnosis on parents’ desire for more children was variable, with half of them (12/24) reporting a change in desire following the diagnosis, including 55% (6/11) of fathers interviewed. Two-thirds of the cohort (16/24) reported a preference for pursuing prenatal genetic diagnosis in the event of a future pregnancy, with 88% (14/16) reporting they would also consider a termination of pregnancy (TOP) if the diagnosis were confirmed. Seventy-three percent (8/11) of fathers interviewed were in favor of a TOP compared to 46% (6/13) of mothers.

*Father: “The topic is closed, because even if there is a 25% risk of it happening again, we consider it a selfish risk to take. I would never do it again, even if the risk were 0.1%, in the interest of the child firstly, and for us as parents”.*

The most frequently cited reasons for not wanting more children were (i) the fear of having another affected child (7/24) and (ii) having insufficient resources to care for another child in the face of their affected child’s already significant needs and uncertain prognosis (6/24).

On the other hand, among respondents still open to having more children, one mother described her inner conflict at the prospect of having another affected child and alluded to a change of perspective.

*Mother: “Before my child was born, we were certain that if anything had come up on prenatal screening, we would have opted for an abortion. But now that she is born, we have doubts about what is right or wrong for us. My opinion of what matters has changed, and she is no less of a human…” [interrupted because of crying]*

## 4. Discussion

This study explored the value placed on a genetic diagnosis by parents of children with PMDs and sought to gain broader insight into its impacts on their lived experience. After interviewing 24 parents, their psychosocial experiences were categorized according to the following major themes: (1) the emotional value of a PMD diagnosis, (2) the challenges of navigating the healthcare system, (3) changes in daily life and relationship dynamics within the nuclear family, (4) the values of social support, and (5) the impact on family planning. Understanding their experiences beyond the diagnosis helps to highlight important supportive care needs.

The emotional impact of a genetic diagnosis in a child with a PMD was often experienced by parents as a mix of relief and disappointment, both of which could be felt either concurrently or separately over the course of their journeys. Being able to name the mitochondrial disease provided a valuable sense of validation for parents and in some cases also reduced their sense of guilt. This is similarly reflected in studies focusing on the value that parents place on the diagnostic etiology of intellectual disability or rare diseases [10,22]. Given the variable nature of any mitochondrial disorder, however, and the lack of formal treatment guidelines, a clinical diagnosis may not fulfill parental expectations of clarity around prognosis and treatment options [23]. The ongoing prognostic uncertainty and lack of curative therapies for PMDs were often cited by parents as drivers for ongoing worry, sadness, and loss of hope. Greater rates of stress, anxiety, and depression have been reported in caregivers of children with PMD compared to parents of children affected by other chronic conditions or inborn errors of metabolism [14,15,17,18,24,25]. Overall, our findings are consistent with previous research describing similar mixed emotions in parents following a rare disease diagnosis in children [8,9,11,26]. Parents highlighted the importance of honest and empathic communication by doctors during the diagnostic disclosure and ongoing follow-up. Furthermore, because emotional responses can impact one’s ability to process information, parents also appealed for the provision of written information and reliable online resources that cater to different language backgrounds and are written in non-technical language.

Many parents felt that a PMD diagnosis justified their desire to pursue more intensive therapy regimes for their child. Accordingly, another study found that parents of children with rare disorders diagnosed by exome sequencing felt that the diagnosis led to more focused medical management for their child [9] Unexpectedly, however, most parents in our study also felt that a PMD diagnosis did not fundamentally change access to supportive therapies or medical equipment for their child. This dissonance between parents’ motivation and their ability to access support appears to be multifactorial, related in part to the financial burden of a PMD diagnosis, as well as the challenges these families face in successfully navigating the healthcare system to advocate for their child’s needs. Indeed, many of the parents interviewed felt overwhelmed and unsupported. This is in keeping with the previous literature showing that families of children affected by PMDs often experience high levels of financial and caregiver burden [12,14,19,24]. Parents in our study highlighted a need for better health insurance subsidies and improved access to supportive therapies, including physiotherapy and psychological support. Furthermore, there was a strong demand for social workers to provide practical support by sourcing relevant services, applying for healthcare benefits, and advocating for a child’s needs.

In many families, confirmation of a PMD diagnosis led to a significant shift in attention towards the affected child. The distribution of caregiving responsibilities remained unchanged in most of the families we interviewed, with mothers often assuming a greater proportion of the responsibility. Despite participants’ emphasis on the importance of supporting each other in parenthood, a PMD diagnosis had ensuing consequences on relationship dynamics between other members of the nuclear family. Increased marital strain was common, and some parents also shared concerns about the adverse impact of a diagnosis on unaffected siblings. Similar changes in spousal and sibling dynamics have been described in families of children affected by other inborn metabolic diseases [25,27], and it seems plausible to speculate about the possible influence of financial and psychosocial stressors associated with the high burden of care in these conditions. Parents highlighted a need for additional in-home care and respite services to help reduce the burden of care and improve their quality of life. Improved access to psychological support, including couples counseling or family therapy, could assist with improving communication styles and help to preserve a stable parent partnership.

All participants acknowledged the important role of family and social support in helping them cope with their child’s diagnosis. The optimal sources of this support were highly variable, however, highlighting each family’s unique set of circumstances and the different challenges they face in connecting with others. This is in keeping with the findings of Senger et al. who explored some of the coping strategies used by parents of children with mitochondrial disorders [19]. Interestingly, all respondents in that study were active members of social media platforms known to the mitochondrial community, whereas most of the parents that we interviewed relied more heavily on pre-existing networks of family and friends for support. In some cases, the phenotypic variability within any given PMD diagnosis and differences in children’s developmental trajectories hampered some parents’ willingness to connect with other affected families through disease-support groups, a finding similar to that reported for other genetic conditions [26]. Rajasekar et al. also evaluated the psychosocial stressors and support in parents of children with inborn metabolic diseases [25]. Parents in that study described changes in friendship trajectories occurring in their lives post-diagnosis, with new friendships being forged and some pre-established relationships becoming eroded over time due to low disease awareness or perceived discomfort. In our study limited disease awareness within respondents’ own friendship circles or extended family also contributed to communication challenges and led some of the parents we interviewed to limit those interactions or isolate. Regardless of the sources of support post-diagnosis, parents’ responses highlighted a yearning to be seen, heard, and understood, a desire to connect with similarly affected families and to have others bear witness to their lived experience in a safe and trusting environment.

Most participants sought genetic counseling post-diagnosis. The value of family-specific genetic information regarding inheritance risks, prenatal testing options and implications for surviving relatives has also previously been highlighted by parents of children who had died of a PMD disorder [28]. There was a strong preference for prenatal genetic testing in the event of a future pregnancy. Most parents reported favoring a termination of pregnancy if this confirmed the PMD diagnosis, commonly citing the high burden of care involved. A small number of parents remained undecided, and in one mother’s case, a possible explanation for her inner conflict might be that after loving and caring for her child with a PMD, terminating the pregnancy of a similarly affected child became more difficult to envision. The variable responses to family planning and preference for prenatal genetic diagnosis in future pregnancies underscore the importance of genetic counseling to support couples in making a decision that is right for them.

Restricting the inclusion criteria to single-household families allowed us to explore the impact of a child’s PMD diagnosis on the (re)distribution of shared care responsibilities, as well as differences in coping strategies and interactions with extended family and friends between mothers and fathers. The limited number of studies exploring the psychosocial experiences of parents caring for children with PMDs primarily discuss maternal experiences [12,14,15,16]. Conducting separate interviews with parents allowed us to gain greater insight into the paternal perspectives as well. For the most part, responses from mothers and fathers were largely concordant. The fathers’ perspectives differed more prominently from the mothers’ in relation to the extent of emotional relief experienced after diagnostic confirmation in their child, as well as their desire not to have more children in the future because of such a diagnosis. Other differences relating to the perceived impacts of a diagnosis on the structure of everyday life, financial changes, or on perceived benefit derived from various support networks were less marked. Ultimately, the small cohort of 13 mothers and 11 fathers interviewed makes it difficult to draw robust conclusions regarding gender-based differences and warrants larger multicenter studies to investigate this further.

There are important study limitations to acknowledge. Participants were recruited from a single tertiary center in Austria and only those fluent in German and still living together were included, thereby affecting sample size and limiting the generalizability to broader international communities. Our study highlighted changes in family dynamics post-PMD diagnosis, including increased marital strain. By solely focusing on single-household families, however, this study did not capture the experiences of families fractured due to carer burden. In one previous study using an internet survey to assess stressors and coping of parents caring for a mitochondrial disease, 45/230 (19%) respondents were classed as single/divorced/widowed [19]. Moreover, all participants were Caucasian and mostly well-educated, so findings may not be generalizable to other patient groups. In particular, the unique challenges faced by families of culturally and linguistically diverse backgrounds in navigating the healthcare system are likely to be under-represented [22]. Including this demographic in future research will be key to providing a more complete depiction of the spectrum of parents’ psychosocial experiences in caring for a child with PMD. While there are likely to have been additional psychosocial experiences that were not captured due to the study’s strict inclusion criteria, focusing on native German speakers was an attempt to ensure a more homogeneous cohort, to potentially reduce the confounding influence of any language barrier on parental experiences. Furthermore, one could also argue that it facilitated capturing some of the more subtle aspects of participants’ psychosocial experiences that might have otherwise been lost in translation using an interpreter. Additionally, because interviews with participants were only carried out at a single point in time, we could not ascertain if and how parents’ lived experience and perceived impact of a diagnosis changes over time. The timing of interviews relative to a child’s diagnosis ranged up to 7 years post-diagnosis, adding to the heterogeneity of responses. The interview questionnaire that was used was not tested for its reliability or validity, and results should therefore be interpreted with caution. Finally, this study did not address the experience of parents whose children remain undiagnosed despite high suspicion of a PMD, and who likely have to contend with the effects of this added layer of uncertainty. It is possible, nonetheless, that they share similar experiences to those of parents we interviewed, as previously described in parents of children with or without a known cause of intellectual disability and for whom descriptive labels such as “epilepsy” or “autism” were more valuable than a precise diagnosis for securing services for their children [10].

## 5. Practice Implications and Future Directions

This study provides valuable insight into the psychosocial challenges and lived experiences of parents following a genetic diagnosis of a PMD in their child. Understanding their experiences beyond the diagnosis is important to highlight gaps and draw awareness to the supportive care needs of families affected by PMDs. Included in this are a constellation of emotional, psychological, informational, practical, and social needs, as well as others [29]. More research, including larger multicenter studies, is needed to explore how parents’ psychosocial experiences and needs evolve at different stages of the child’s development. These studies serve as an important reminder to healthcare workers that focusing on providing a molecular diagnosis may overlook the larger journey parents are often on—the therapeutic odyssey, a search for “anything that will help their child, walk, talk, run, play, learn to read and write and wonder and search for answers” [30,31].

*Father: “The diagnosis was not an end point; it was another starting point in and of itself”.*

## Figures and Tables

**Table 1:** Demographic characteristics.

Parents (N = 24)	Mothers (N = 13)	Fathers (N = 11)
Median age, years [range]	44 [29–54]	46 [39–57]
Highest level of education attained		
Secondary school	1	0
Apprenticeship	5	5
High school diploma	2	2
University degree	5	4
Average paid employment hours per week, mean [SD]	20.5 [6.4]	40.4 [1.7]
Gross monthly income, n		
Unemployed	3	0
<EUR 1000	1	0
EUR 1000–2999	9	2
EUR 3000–4999	0	3
>EUR 5000	0	6
Average yearly household income (gross)	EUR 59,720	
Median yearly household income (gross)	EUR 60,000	
Children (N = 13)		
Sex	Males 4; Females 9	
Median age at symptom onset, years [range] ^$^	1 [0–15]	
Median age at diagnosis, years [range] ^$^	4 [0.08–19]	
Median diagnostic odyssey, years [range] ^$^	1.5 [0.08–19]	
Median age at parent enrolment, years [range]	9 [2–19]	
Median timing of interview since diagnosis, years [range]	2 [0–7]	
Molecular etiologies identified		
Nuclear DNA defects	N = 10	
Affected gene (variant zygosity, inheritance)	*CLPB* (MA, DN) (N = 2)	
	*ATP5F1E* (BA, I)	
	*NUBPL* (BA, I)	
	*TRMU* (BA, I)	
	*BCS1L* (BA, I)	
	*C1QBP* (BA, I)	
	*TK2* (BA, I)	
	*PDHA1* (XL, DN)	
	*FARS2* (BA, I (x1) and suspected DN (x1))	
Mitochondrial DNA defects	N = 3	
Variant, gene (tissue-specific heteroplasmy)	m.8993T>C, *MT-ATP6* (Bl 100%; mother Bl 14%)	
	m.3243A>G, *MT-TL1* (Bl 35%, Bu 61%, U92%)	
	m.10191T>C, *MT-ND3* (M 67%, Bl 48%, U 74%)	

**^$^** as reported by parents. BA, biallelic; MA, monoallelic; XL, X-linked; DN, de novo; I, inherited; Bl, blood; Bu, buccal; M, muscle; U, urine.

**Table 2:** Thematic analysis.

		Mothers	Fathers	Total (%)
**Emotional value of a genetic PMD diagnosis**				
Diagnosis provided validation		10/13	7/11	17/24 (71)
Diagnosis was empowering		11/13	8/11	19/24 (79)
Diagnosis provided relief		9/13	3/11	12/24 (50)
Diagnosis helped talk to friends/family about condition		10/13	10/11	20/24 (83)
**Challenges of navigating the healthcare system**				
Diagnosis justified need for supportive therapies		10/13	9/11	19/24 (79)
Diagnosis did not change access to supportive therapies		9/13	7/11	16/24 (67)
Insufficient subsidies for supportive therapies despite a diagnosis		9/13	7/11	16/24 (67)
**Changes in daily life and relationship dynamics within the nuclear family**				
Diagnosis did not change distribution of caregiving activities		9/13	7/11	16/24 (67)
Caregiving is compatible with keeping paid employment		8/13	8/11	16/24 (67)
Diagnosis led to changes structure of daily life		7/13	4/11	11/24 (46)
Diagnosis impacted finances		7/13	5/11	12/24 (50)
Diagnosis altered relationship dynamics in nuclear family		7/13	8/11	15/24 (63)
**Value of social support**				
Diagnosis changed interactions with extended family		8/13	5/11	13/24 (54)
Diagnosis did not change interactions with friends		10/13	7/11	17/24 (71)
Family and friends are a source of relief		10/13	8/11	18/24 (75)
Diagnosis did not change contact with community support groups		10/13	10/11	20/24 (83)
Community support groups are a source of relief		7/13	5/11	12/24 (50)
**Family Planning**				
Genetic counseling sought post diagnosis		12/13	10/11	22/24 (92)
Diagnosis changed desire for more children		6/13	6/11	12/24 (50)
Preference for prenatal genetic testing		8/13	8/11	16/24 (67)
Preference for termination of pregnancy if PMD confirmed		6/13	8/11	14/24 (58)

## Data Availability

The raw data supporting the conclusions of this article will be made available by the authors on reasonable request.

## Supplementary Materials

The following supporting information can be downloaded at: https://www.mdpi.com/article/10.3390/jpm14050523/s1, Table S1: Semi-structured interview questions.
