# Supplementary material for: Ending an Odyssey? The Psychosocial Experiences of Parents after the Genetic Diagnosis of a Mitochondrial Disease in Children
Source: J Pers Med. 2024 May 14;14(5):523. doi: 10.3390/jpm14050523 (PMC11122152; doi:10.3390/jpm14050523)
Supplement: Supplementary file 1 [file jpm-14-00523-s001.zip › jpm-2983046-supplementary.pdf]

Table S1. Semi-structured interview questions

**Demographic information**	**Interview Questions**
**Parent**	Your gender is □ male □ female □ diverse
	You are _____ years old
	Your highest educational qualification is □ Secondary school □ Apprenticeship □ High School (Abitur/Matura) □ University degree
	Paid working hours/week:
	Your average monthly income is (gross income) □ < 1,000 € □ up to 1,500 € □ up to 2,000€ □ up to 3,000€ □ up to 4,000€ □ up to 5,000€ □ > 5,000€
	You have □ 1 child □ 2 children □ 3 children □ 4 children □ > 4 children
**PMD-affected child**	Your child is □ male □ female □ diverse
	Your child is ____ years old
	How old was your child when the first symptoms appeared?
	How old was your child at diagnosis?

**Topic**	**Interview Questions**
**Diagnosis experience**	Did the diagnosis help to classify the symptoms as a disease?
	Did the diagnosis change the way you deal with the disease?
	Did the diagnosis provide you emotional relief?
	What feelings or thoughts arose for you in the moment of receiving the diagnosis?
	What else would have been helpful for you in the phase of getting the diagnosis?
**Daily life and lifestyle after diagnosis**	Did the diagnosis change the way you structure your everyday life?
	Did the division of caregiving responsibilities between parents changed after diagnosis?
	Was it still possible for you to maintain paid employment?
	Did the diagnosis impact your financial situation?
	Were there any other changes that resulted from the diagnosis (e.g. vacation planning or your leisure activities)?
	What else would have been helpful for you in structuring your everyday life?
**Access to therapeutic supports (i.e. services and equipment)**	Did the diagnosis justify for you the need for therapies or eligibility for therapeutic supports?
	Did the diagnosis change your access to therapeutic supports?
	Was access to therapeutic supports subsidised as a result of the diagnosis?

	Which therapies or aids were particularly relieving for you?
	What else would have been helpful for you regarding therapeutic supports?
**Support groups and other affected families**	Did the diagnosis affect your decision to access to disease-specific support groups or contact with other affected families?
	Did you find that participation in community support groups or contact with other affected families provided support or relief?
	What topics in the social exchange did you find useful or relieving?
	What else would have been helpful for you regarding interactions with other affected families or support groups?
**Interaction with family members and friends**	Did the diagnosis affect interactions within your nuclear family?
	Did the diagnosis affect interactions with extended family members or friends?
	Did the diagnosis help you to talk about the disease or to be able to explain it better to others?
	Did you find that interactions with family and friends provided support or relief?
	What else would have been helpful regarding interaction with family members and friends?
**Family planning**	Did you seek genetic counselling regarding genetic risk for siblings and recurrence risk after diagnosis?
	Did the diagnosis change your desire to have more children?
	Would you use prenatal genetic diagnosis for an early clarification in a future pregnancy?
	Would you consider an abortion based on prenatal evidence of a mitochondrial disease?
	What else would be helpful for you regarding family planning?
**Closing question**	What else would have been helpful for you from our clinic?
